# ncRNAs-mediated high expression of TIMM8A correlates with poor prognosis and act as an oncogene in breast cancer

**DOI:** 10.1186/s12935-022-02595-x

**Published:** 2022-05-02

**Authors:** Zhonglin Wang, Shuqin Li, Feng Xu, Jingyue Fu, Jie Sun, XinLi Gan, Chuang Yang, Zhongqi Mao

**Affiliations:** 1 Department of General Surgery, The First Affiliated Hospital of Soochow University, Soochow, 215006 China; 2 Department of Breast Surgery, The Second People’s Hospital of Lianyungang, Lianyungang, 222006 China; 3 Department of Breast Surgery, The Affiliated Lianyungang Hospital of Xuzhou Medical University, Lianyungang, 222006 China; 4 Jiangsu Breast Disease Center, The First Affiliated Hospital With Nanjing Medical University, Nanjing, 210029 China

**Keywords:** TIMM8A, ncRNA, Prognosis, Proliferation, Breast cancer

## Abstract

**Background:**

Breast cancer is notorious for its increasing incidence for decades. Ascending evidence has demonstrated that translocase of inner mitochondrial membrane (TIMM) proteins play vital roles in progression of several types of human cancer. However, the biological behaviors and molecular mechanisms of TIMM8A in breast cancer remain not fully illustrated.

**Methods:**

Pan-cancer analysis was firstly performed for TIMM8A’s expression and prognosis by Oncomine database. Subsequently, TIMM8A-related noncoding RNAs (ncRNAs) were identified by a series of bioinformatics analyses and dual-luciferase reporter assay, including expression analysis, correlation analysis, and survival analysis. Moreover, the effect of TIMM8A on breast cancer proliferation and apoptosis was evaluated in vitro by CCK-8 assays, EdU cell proliferation assays, JC-1 mitochondrial membrane potential detection assays and Western blot assays and the in vivo effect was revealed through a patient-derived xenograft mouse model.

**Results:**

We found that TIMM8A showed higher expression level in breast cancer and the higher TIMM8A mRNA expression group had a poorer prognosis than the lower TIMM8A group. hsa-circ-0107314/hsa-circ-0021867/hsa-circ-0122013 might be the three most potential upstream circRNAs of hsa-miR-34c-5p/hsa-miR-449a-TIMM8A axis in breast cancer. TIMM8A promotes proliferation of breast cancer cells in vitro and tumor growth in vivo.

**Conclusion:**

Our results confirmed that ncRNAs-mediated upregulation of TIMM8A correlated with poor prognosis and act as an oncogene in breast cancer.

**Supplementary Information:**

The online version contains supplementary material available at 10.1186/s12935-022-02595-x.

## Introduction

Breast cancer is the most frequently diagnosed cancer and one of the leading causes of death among women worldwide [1]. Most breast cancer patients are usually diagnosed at an advanced stage, with a poor prognosis. At present, the main treatment of breast cancer is adjuvant chemotherapy and radiotherapy after surgical resection and the specific treatment depends on its subtype and the overall condition of the individual patient. Despite advances in the diagnosis and treatment of breast cancer in recent years, the 5-year survival rate remains unsatisfactory. Therefore, it is important to validate innovative and effective diagnostic biomarkers and therapeutic targets for the treatment of breast cancer.

The function of mitochondrial is integral to eukaryotic cellular metabolism [2] and mitochondria play key functions in cell physiology, including generation of reactive oxygen species, adenosine triphosphate, calcium homeostasis and apoptosis [3, 4]. The metabolically dysfunctional mitochondria are emerging as a hallmark of cancer pathogenesis and result in the different metabolic rate between breast cancer and normal breast tissue [5–7]. Actually, mitochondria have a pivotal impact on virtually all procedures about oncogenesis, including tumor progression, malignant transformation, anticancer immunosurveillance and response to treatment [8, 9]. Proteins which constitute transporting channels in the mitochondrial inner membrane are referred to as translocase of inner mitochondrial membrane proteins (TIMM) [10]. The function of mitochondria is dramatically correlated with the protein-transporting system of the inner mitochondrial membrane including TIMM [11].

In recent years, the clinical importance of TIMM family has been increasingly emphasized [12]. Studies by both Salhab et al. [2] and Cai et al. [13] have revealed that TIMM17A expression was significantly associated with tumor grade, nodal positivity, and stage, as well as with overall and disease-free survival of breast cancer. Wang et al. [14] suggested that TIMM9 expression was augmented in gastric tumor tissues compared with paired normal gastric tissues. TIMM8A, as a translocase of inner mitochondrial membrane, is involved in the import and insertion of hydrophobic membrane proteins from the cytoplasm into the mitochondrial inner membrane [15]. The mutation in the TIMM8A gene could cause deafness‐dystonia‐optic neuronopathy syndrome [16]. However, no study has evaluated functional role of TIMM8A in cancer.

In the present study, we first performed expression analysis and survival analysis for TIMM8A in multiple types of human cancer. Next, the noncoding RNA (ncRNA)-associated regulation of TIMM8A, involving microRNAs (miRNAs) and circular RNAs (circRNAs), was also predicted in breast cancer. Finally, we explored the biological functions of TIMM8A in breast cancer cell lines (MCF7 and MDA-MB-231) and patient-derived tumor fragment platform. Taken together, our findings reveal that ncRNAs-regulated TIMM8A correlates with poor prognosis of patients in breast cancer and acts as an oncogene in breast cancer.

## Materials and methods

### Patients and samples

The breast cancer clinical samples involved in this experiment were obtained from the Second People’s Hospital of Lianyungang in 2021. This study was approved by the hospital ethics committee, and samples were obtained upon informed consent from the patients.

### TCGA data download, process and analysis

The TIMM8A mRNA expression data of breast cancer was downloaded from The Cancer Genome Atlas (TCGA) database (https://tcga-data.nci.nih.gov/tcga/). These data were normalized and then analyzed for differential expression using limma R package.

### ONCOMINE data-mining analysis

Oncomine database (https://www.oncomine.org/) is a public online cancer database for RNA and DNA sequences. In our study, firstly, it was used to analyzed the transcriptional levels of TIMM8A in 20 types of human cancer. Then, we compared the mRNA expression of TIMM8A in breast cancer samples with those in normal samples using Students t-test. Statistically significant values and fold change were defined as p-value ≤ 1E-4, and 2, respectively.

### GEPIA database analysis

GEPIA database (http://gepia.cancer-pku.cn/) is a web database for differential expression analysis based on TCGA and The Genotype-Tissue Expression (GTEx) data. GEPIA was utilized to discover TIMM8A expression in breast cancer. GEPIA was employed to perform survival analysis for TIMM8A in breast cancer, including overall survival (OS) and relapse-free survival (RFS). p value < 0.05 was considered as statistically significant.

### Kaplan–meier plotter

The Kaplan Meier Plotter (http://kmplot.com/analysis/) provides survival information for patients with breast cancer. We used it to reveal the clinical relationships between miRNAs expression and survival information including RFS and OS. The prognostic value of upstream binding miRNAs of TIMM8A, such as p-values, hazard ratios (HR), and 95% confidence intervals could be automatically calculated on the basis of the RNA expression (high vs. low) of miRNAs.

### Candidate miRNAs and circRNAs prediction

Upstream binding miRNAs and circRNAs of TIMM8A were predicted by several target gene prediction databases. miRNAs targeting TIMM8A were predicted by starBase (http://starbase.sysu.edu.cn/) and TargetScan (http://www.targetscan.org/vert_72/). The Cancer-specific circRNAs database (CSCD) [17] and circbank (http://www.circbank.cn/index.html) were utilized to predict target circRNAs. Overlapped circRNAs in the two databases were considered as potential target ciriRNAs of miRNAs.

### Functional enrichment analysis

KEGG pathway enrichment and GO functional enrichment were respectively implemented by the KEGG database (http://www.genome.jp/kegg/) and GO database (http://geneontology.org). The statistical significance was examined via the hypergeometric test, and p < 0.05 was considered the threshold to identify significantly enriched KEGG pathways and GO terms as reported [18]. GSEA tools (http://www.broadinstitute.org/gsea) were used to analyze the potential biological processes and pathways regarding to TIMM8A [19]. And the STRING database (http://string-db.org, access date 30 July, 2021) was employed to predict protein–protein interactions (PPI) and constructing the PPI network.

### Cell culture and transfection

Human breast cancer cell lines MCF-7 and MDA-MB-231 were purchased from American Type Culture Collection (ATCC). HEK-293T cell line was preserved by our lab. Cells were cultured in Dulbecco’s modified eagle medium (DMEM) (Gibco, USA) containing 10% fetal bovine serum (Wisent, Canada), 100 U/ml penicillin and 100 mg/ml streptomycin. Cells were cultured and maintained in an incubator containing 5% CO_2_ at 37 °C. siRNA (GenePharma, China) was used to knockdown TIMM8A. Cells were grown to 80–90% confluency at the time of transfection. Cells were transfected with siRNA by Lipofectamine^®^ 3000 reagent (ThermoFisher Scientific, USA) and 72 h after transfection, cells were harvested to extract total RNA and protein. qRT-PCR and western blot were used to detect the transfection efficiency.

### quantitative RT-PCR

Total RNA was extracted from the cells or tissues by using Trizol Reagent (TaKaRa, Japan). cDNA synthesis was performed using the HiScript qRT SuperMix (Vazyme, China). AceQ qPCR SYBR Green Master Mix (Vazyme, China) was used for qRT-PCR in a real-time PCR instrument (Roche, USA) by manufacturer’s instructions. β-actin was used as the endogenous control. The sequences of primers used are as follows: β-actin: forward 5′-GCTGTGCTATCCCTGTACGC-3′ and reverse 5′-TGCCTCAGGGCAGCGGAACC-3′; TIMM8A: forward 5′-CAGCATTTCATCGAGGTAGAGAC-3′ and reverse 5′- AGCCCGACTGTCCAACTTTG-3′; Ki67: forward 5′-GACCTGTTCTTTGAGGCTGAC-3′ and reverse 5′-TCCATCTTCTTCTTTGGGTATTGTT-3′.

### Western blot analysis

The total protein was extracted from cells with RIPA buffer (Beyotime, China) containing phenylmethylsulphonyl fluoride (PMSF), protease inhibitors and phosphatase inhibitors (Beyotime, China). Total protein was separated by sodium dodecyl sulfate polyacrylamide gel electrophoresis (SDS-PAGE) by a 10% SDS-PAGE gel, and then transferred onto a polyvinylidene difluoride (PVDF) membrane (Millipore, USA). The membranes were blocked with 5% skimmed milk powder in Tris buffered saline containing 0.1% Tween 20 (TBST) for 2 h at room temperature and probed with primary antibody against TIMM8A, Bax, Bcl-2 and GAPDH (CST, USA). The membranes were then washed three times with TBST, and incubated with the appropriated secondary antibodies for 2 h. Finally, the bands were examined through Immobilob™ Western Chemiluminescent HRP Substrate (Millipore, USA) to detect the expression levels of target proteins.

### Cell counting kit (CCK-8) assay

Cell proliferation was determined using CCK-8 kit (Dojindo, Japan) according to the instructions of the manufacturer. 3000 cells were suspended in 200 μl medium and seeded in triplicate in a 96-well plate, grown in an incubator containing 5% CO_2_ at 37 °C overnight. The original medium was replaced by medium containing 10% CCK8 and then incubated at 37 °C for 2 h. Next, a microplate reader was determined the absorbance at 450 nm.

### EdU assay

EdU assay was performed using an EdU assay kit (RiboBio, China). In short, cells were seeded in 96-well plates (1 × 10^4^ cells/well) overnight and transfected with control siRNA and TIMM8A siRNA for 72 h. After incubated with EdU (50 µM) for 2 h, the samples were fixed in 4% formaldehyde for 15 min. Subsequently, cells were permeabilized with 0.3% TritonX-100 (Beyotime, China) for 10 min and then reacted with Apollo reaction mixture for 30 min. Nuclei were stained with DAPI (Beyotime, China) for 15 min and images were taken by a fluorescent microscope (Nikon, Japan).

### JC-1 mitochondrial membrane potential detection assay

JC-1 assay was performed to measure mitochondrial membrane potential (ΔΨm) using the JC-1 mitochondrial membrane potential assay kit (KeyGen, China). In short, cells were seeded in 6-well plate and 72 h after siRNA transfection, the detection of ΔΨm was carried out according the guidelines of JC-1 kit. Images were taken by a fluorescent microscope (Nikon, Japan).

### Dual-luciferase reporter assay

Dual-luciferase reporter assay was used to illustrate the targeting binding relationship between miRNA and the mRNA of TIMM8A in HEK-293T cells. In short, wild-type or mutant TIMM8A 3′UTR dual-luciferase reporter plasmid (Gene Pharma, China) and hsa-miR-34c-5p mimics or mimics control (RiboBio, China) were co-transfected into HEK-293T cells using the Lipofectamine 3000 reagent (Invitrogen, USA). After 48 h of transfection, the activities of Firefly and Renilla luciferase were determined according to the manufacturer’s instructions.

### Ex vivo tumor fragment platform

The patient-derived tumor fragment platform was constructed as previously described [20]. Briefly, fresh tumor tissue from surgical resections was dissected into fragments of approximately 1 mm^3^. The fragments were embedded into an artificial extracellular Matrigel matrix (BD Biosciences, USA) and then seeded in 24-well plates. The ex vivo tumor fragment platform was cultured using organoid growth medium (TEMCELL™ Technologies, Canada) and maintained in an incubator containing 5% CO_2_ at 37 °C.

### Patient-derived xenograft model

The patient-derived xenograft (PDX) model was carried out as previously described [21]. Briefly, we implanted breast cancer samples of 75 mm^3^ into the fourth mammary fat pad of severely immunocompromised NOD/SCID mice (aged 4 weeks, 18–22 g) and estradiol were added to the mice using patches. Tumor volume was measured every 3 days using a caliper calculated as (length width height)/2. One week after implantation, PBS or TIMM8A inhibitor was intra-tumor injection every 3 days until mice were sacrificed. Euthanasia was performed by cervical dislocation after induction of terminal anesthesia using 5% isoflurane which was in accordance with the American Veterinary Medical Association (AVMA) Guidelines: for the Euthanasia of Animals (2020 Edition). All animal experiments were approved by the Animal Use Committee of the Nanjing Medical University.

### Statistical analysis

The data were presented as mean ± standard error of the mean (SEM) and analyzed using the GraphPad Prism (Version 8.0) and SPSS 20.0 software. The relationship between TIMM8A expression and clinicopathologic features was analyzed by Pearson chi-square test or Fisher exact test. The univariate and multivariate analysis of the effect of each variable on survival was performed using cox proportional hazards regression model. All experiments were least repeated in triplicate, unless otherwise specified and p < 0.05 was considered as a threshold to indicate a statistical significance.

## Results

### TIMM8A was elevated in human breast cancer cells and tissues

**Fig. 1:**
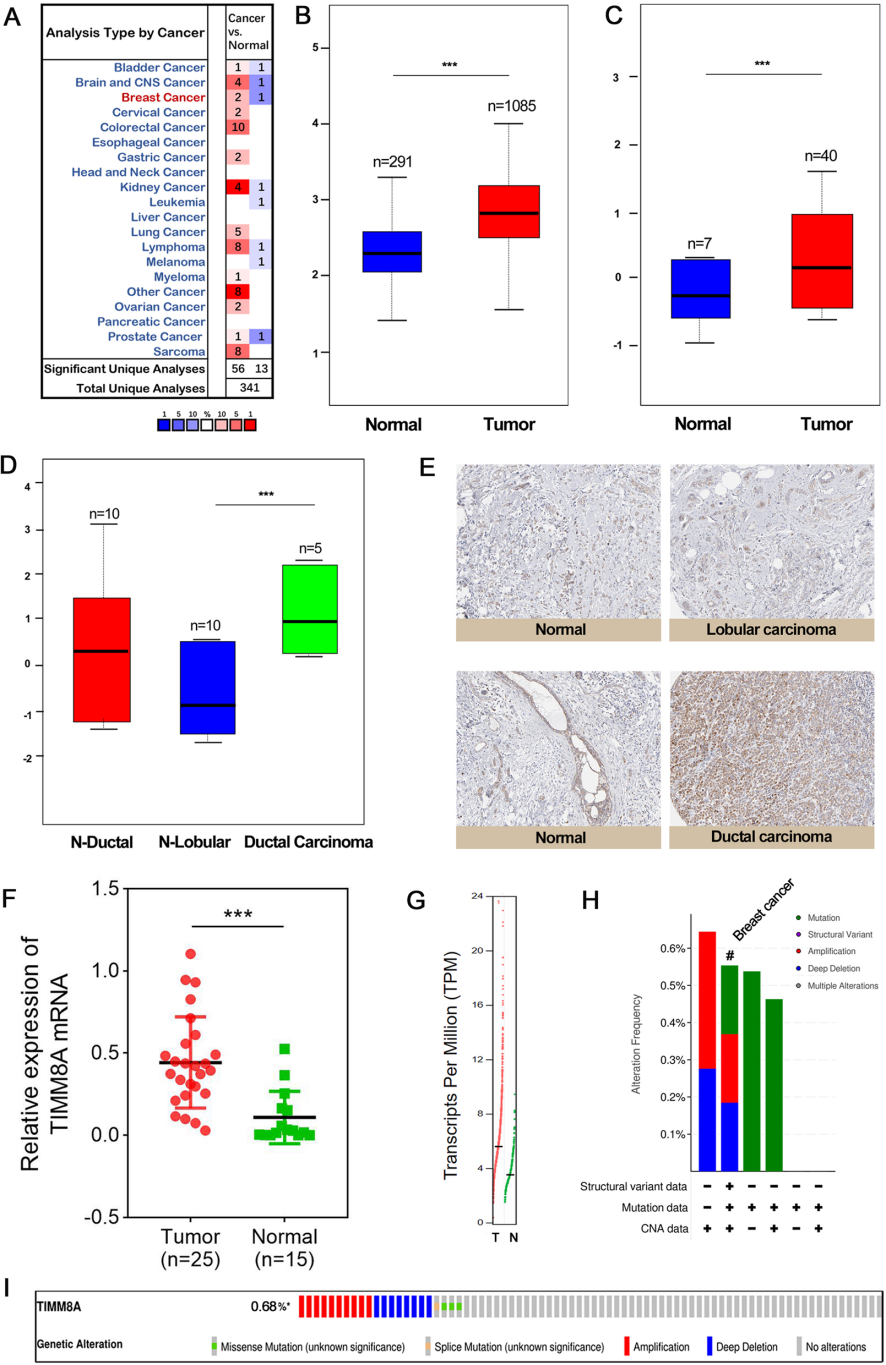
The expression of TIMM8A in breast cancer. **A** The pan-cancer expression profile of TIMM8A in oncomine database. **B** The expression of TIMM8A in normal and tumor tissues of breast in TCGA and GTX data (***p < 0.001). **C** The expression of TIMM8A in Richardson Breast data from oncomine database (***p < 0.001). **D** The expression of TIMM8A in Turashvili Breast data from oncomine database (***p < 0.001). **E** The TIMM8A protein level in the Human Protein Atlas database. **F** The expression of TIMM8A mRNA in 25 breast cancer tissues and 15 normal breast tissues (***p < 0.001). **G** The expression profile of TIMM8A in GEPIA database. **H**, **I** the mutation of TIMM8A in breast cancer from cBioPortal database

We first analyzed the transcriptional levels of TIMM8A in 20 types of human cancer with those in normal samples using Oncomine databases (Fig. 1A). The mRNA expression levels of TIMM8A were significantly upregulated in patients with breast cancer based on TCGA and GTEx database (Fig. 1B). Richardson’s study found that TIMM8A is highly expressed in breast cancer compared with normal tissues (Fig. 1C) [22]. Moreover, the lobular epithelial (N-Lobular) tissues exhibited significantly lower expression of TIMM8A relative to ductal carcinoma (Fig. 1D) [23]. Furtherly, immunohistochemistry (IHC) from the Human Protein Atlas database confirmed the higher expression of TIMM8A in breast cancer ductal carcinoma cells in protein level (Fig. 1E). As presented in Fig. 1F, we further demonstrated that TIMM8A was expressed higher in breast cancer tissues than normal tissues of patients via detecting TIMM8A mRNA from total of 40 samples. In addition, the gene expression profile of TIMM8A between tumor and normal breast tissues was obtained using the GEPIA database (Fig. 1G). An oncoprint plot using the cBioPortal was generated from the data extracted from the TCGA database to analyze and visualize the somatic mutation of TIMM8A. DNA copy number amplifications, mutations, and deep deletion were the main genetic mutations of breast cancer (Fig. 1H). The percentage of TIMM8A genetic alteration among breast cancer patients is 0.68% (74/10950) (Fig. 1I).

### The prognostic values of TIMM8A in breast cancer

**Fig. 2:**
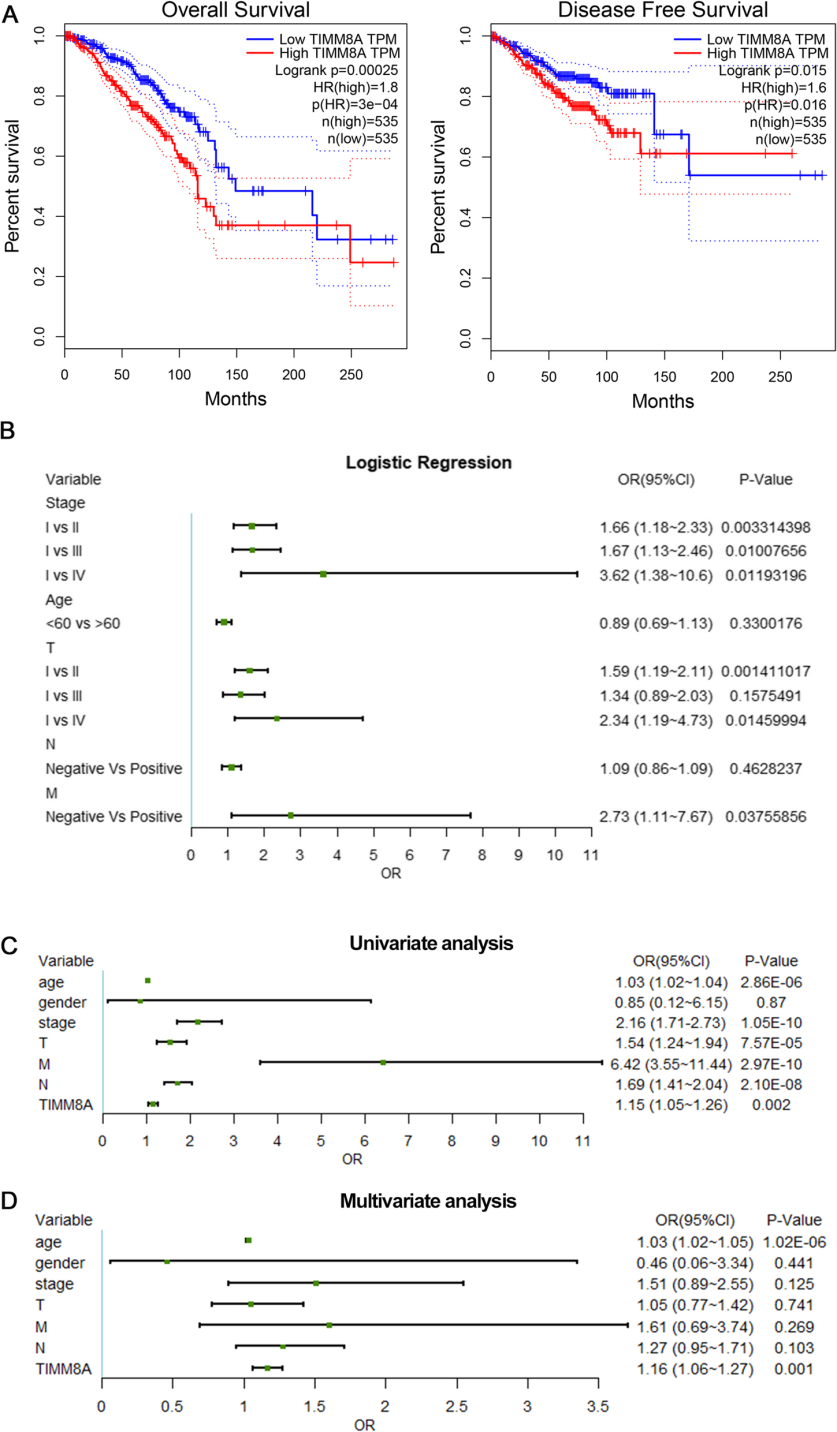
The prognostic values of TIMM8A in breast cancer. **A** The association of TIMM8A mRNA expression and overall survival and disease-free survival was assessed in the GEPIA database. **B** Logistic regression analysis of the association between TIMM8A expression and clinicopathological characteristics in breast cancer patients from TCGA. **C** Univariate analysis of overall survival in the TCGA database. **D** Multivariate analysis of overall survival in the TCGA database

The OS and RFS analysis for TIMM8A in breast cancer determined by the GEPIA database were shown in Fig. 2A, indicating that the higher TIMM8A mRNA expression group has a poorer prognosis than the lower TIMM8A group. By the combination of OS and RFS analysis, TIMM8A may be used as an unfavorable prognostic biomarker in patients with breast cancer. Using logistic regression model, we illustrated the association between TIMM8A and clinicopathological factors in patients with breast cancer from TCGA. As shown in Fig. 2B, TIMM8A expression as a categorical dependent variable (based on a median expression of 25.01) was remarkably associated with stage (I vs II; OR = 1.66, *p* < 0.01), (I vs III; OR = 1.67, *p* < 0.01), (I vs IV; OR = 3.62, *p* < 0.05), T classification (I vs II; OR = 1.59, *p* < 0.05), (I vs IV; OR = 2.34, *p* < 0.05) and metastasis (negative vs positive; OR = 2.73, *p* < 0.05). To identify the prognostic value of TIMM8A in clinical application, we performed the univariate and multivariate cox regression analyses. For TCGA-BRCA data, the TIMM8A in univariate analysis was correlated with OS (OR = 1.15, 95% *CI *1.05–1.26, *p* < 0.01) (Fig. 2C). Multivariate analysis showed that the TIMM8A was an independent prognostic indicator (OR = 1.16, 95% *CI *1.06 1.27, *p* < 0.01) (Fig. 2D).

### Predicted functions and signaling pathways of TIMM8A

**Fig. 3:**
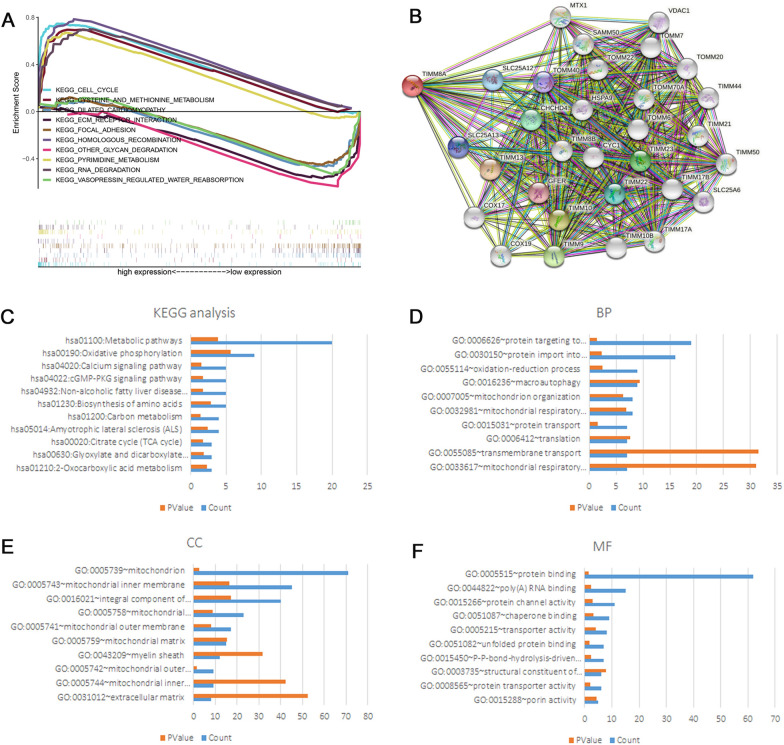
Functional annotation and predicted signaling pathways of TIMM8A. **A** Pathway coexpression network for the top 10 GSEA enriched canonical pathways. **B** Construction and analysis of a PPI network. **C** KEGG enrichment analysis for TIMM8A associated proteins. **D** Biological process (BP) analysis for TIMM8A associated proteins. **E** Cellular component (CC) analysis for TIMM8A associated proteins. **F** Molecular function (MF) analysis for TIMM8A associated proteins

We performed GSEA bioinformatics to analyze the associated biological processes and signaling pathways based on the data acquired from TCGA dataset. Top five upregulated and downregulated pathways were shown in Fig. 3A: including CELL_CYCLE; CYSTEINE_AND_METHIONINE_METABOLISM; DILATED_CARDIOMYOPATHY; ECM_RECEPTOR_INTERACTION; FOCAL_ADHESION; HOMOLOGOUS_RECOMBINATION; OTHER_GLYCAN_DEGRADATION; PYRIMIDINE_METABOLISM; RNA_DEGRADATION; VASOPRESSIN_REGULATED_WATER_REABSORPTION. Additional file 1: Tables S1, S2 show all significantly up- or down-regulated pathways. Thirty outstanding proteins related to TIMM8A were identified by PPI analysis of STRING software (Fig. 3B), such as SLC25A12, TIMM13, COX17, TOMM22, CHCHD4. Kyoto Encyclopedia of Genes and Genomes (KEGG) pathway analysis (Fig. 3C) and Gene Ontology (GO) annotation (Fig. 3D–F) were performed to predict the functions of TIMM8A and the genes dramatically associated with TIMM8A alterations. Metabolic pathways, Oxidative phosphorylation, Calcium signaling pathway and cGMP-PKG signaling pathway are enriched with the most TIMM8 related genes. The complete results of GO and KEGG analyses are shown in Additional file 1: Table S3.

### Prediction and analysis of upstream ncRNAs of TIMM8A

**Fig. 4:**
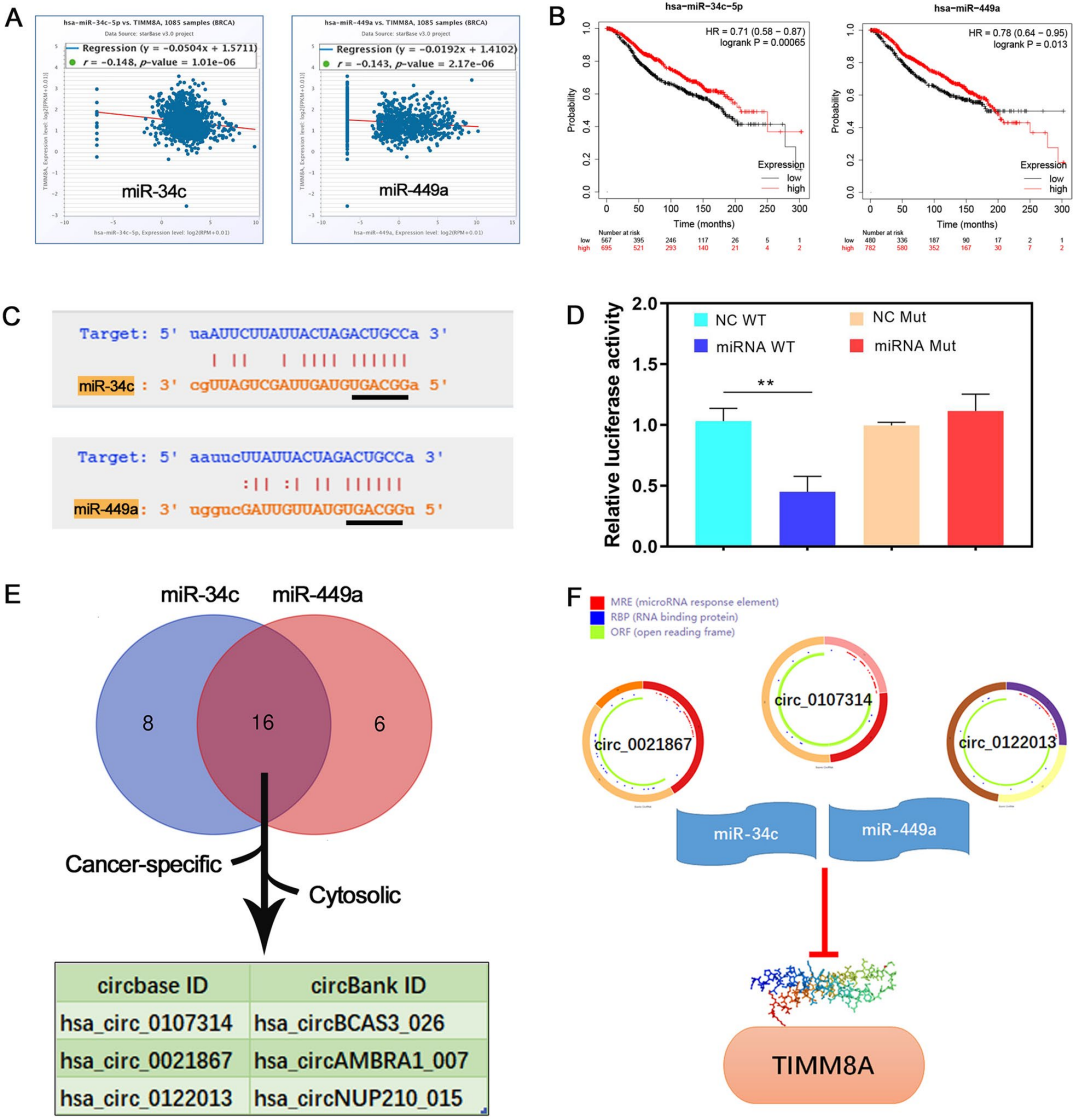
The potential upstream ncRNAs of TIMM8A in breast cancer. **A** The coexpression of miRNAs and TIMM8A in starBase. **B** Kaplan‐Meier curves of breast cancer patients based on hsa-miR-34c-5p and hsa-miR-449a expression using Kaplan‐Meier plotter database. **C** Predicted binding site between hsa-miR-34c-5p and TIMM8A by TargetScan database. **D** The target regulatory relationship was verified using the dual luciferase reporter assay (**p < 0.01). **E** The predicted interacting circRNAs of hsa-miR-34c-5p and hsa-miR-449a. **F** Scheme of ncRNAs-TIMM8A axis

A total of 96 likely target miRNAs of TIMM8A were predicted by starBase (Additional file 1: Table S4). According to the negative correlation principle of miRNA and mRNA of TIMM8A, two candidate miRNAs, hsa-miR-34c-5p and hsa-miR-449a, were selected (Fig. 4A). The correlation between low expression of hsa-miR-34c-5p and hsa-miR-449a and poor prognosis in breast cancer patients was shown in the Kaplan–Meier Plotter database (Fig. 4B). The miRNA target prediction software Targetscan revealed that there was a same binding site (marked with a black line) between hsa-miR-34c-5p and hsa-miR-449a and TIMM8A (Fig. 4C). Furthermore, dual-luciferase reporter gene assay was used to ascertain whether TIMM8A was the direct target gene of hsa-miR-34c-5p and hsa-miR-449a. The results revealed that the relative luciferase activity of TIMM8A-WT was obviously decreased by hsa-miR-34c-5p WT, whereas no similar reduction was observed in the luciferase activity of hsa-miR-34c-5p Mut (Fig. 4D). Moreover, the present study screened the circRNAs that may interact with miRNAs using circBank. 192 candidate circRNAs for hsa-miR-34c-5p and 182 candidate circRNAs for hsa-miR-449a were shown in Additional file 1: Tables S5, S6. Based on conserved sequence, length and binding sites, 24 and 22 potential circRNAs that may interact with hsa-miR-34c-5p and hsa-miR-449a were screened, respectively. Of these, hsa-circ-0107314/hsa-circ-0021867/hsa-circ-0122013 were selected by analyzing their cancer specific property and cytosolic location through Cancer Specific CircRNA Database (Fig. 4E). Taking together, hsa-circ-0107314/hsa-circ-0021867/hsa-circ-0122013 might be the three most potential upstream circRNAs of hsa-miR-34c-5p/hsa-miR-449a-TIMM8A axis in breast cancer (Fig. 4F).

### TIMM8A promote tumor proliferation

**Fig. 5:**
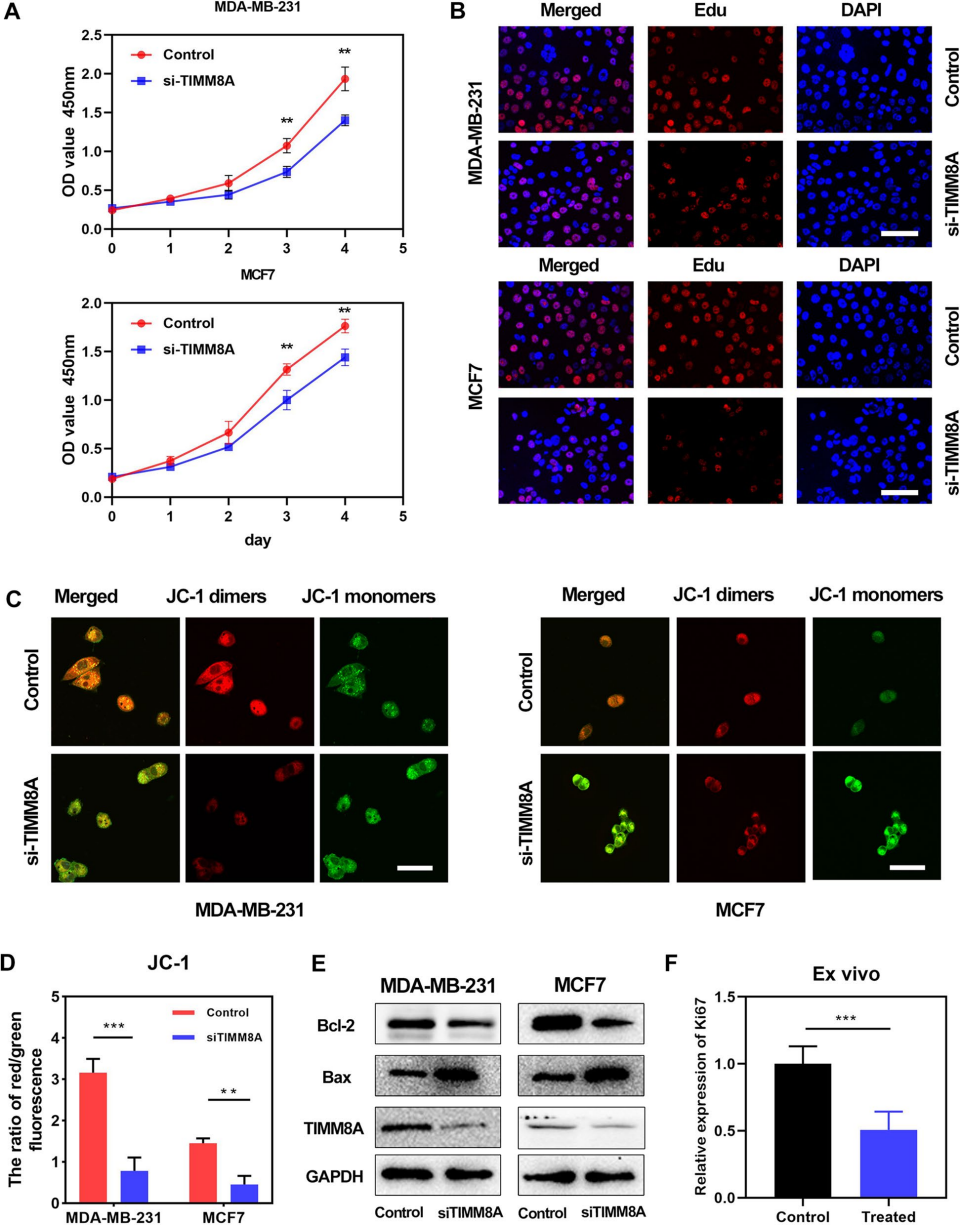
Silencing TIMM8A inhibits the proliferation of breast cancer in vitro. **A** TIMM8A inhibits proliferation of MDA-MB-231 and MCF7 cells by CCK8 assay (**p < 0.01). **B** TIMM8A decreases proliferating MDA-MB-231 and MCF7 cells by EdU assay (Scar bar = 100 µm). **C** The mitochondrial membrane potentials were determined by JC-1 assay (Scar bar = 50 µm). **D** Quantitative analysis of ratio of red/green fluorescent intensity (**p < 0.01, ***p < 0.001). **E** The effect of TIMM8A on apoptosis of breast cancer cells was analyzed by Western blot. **F** The ex vivo model was used to evaluate the effect of TIMM8A on proliferation marker Ki67 in breast cancer tissues (***p < 0.001)

**Fig. 6:**
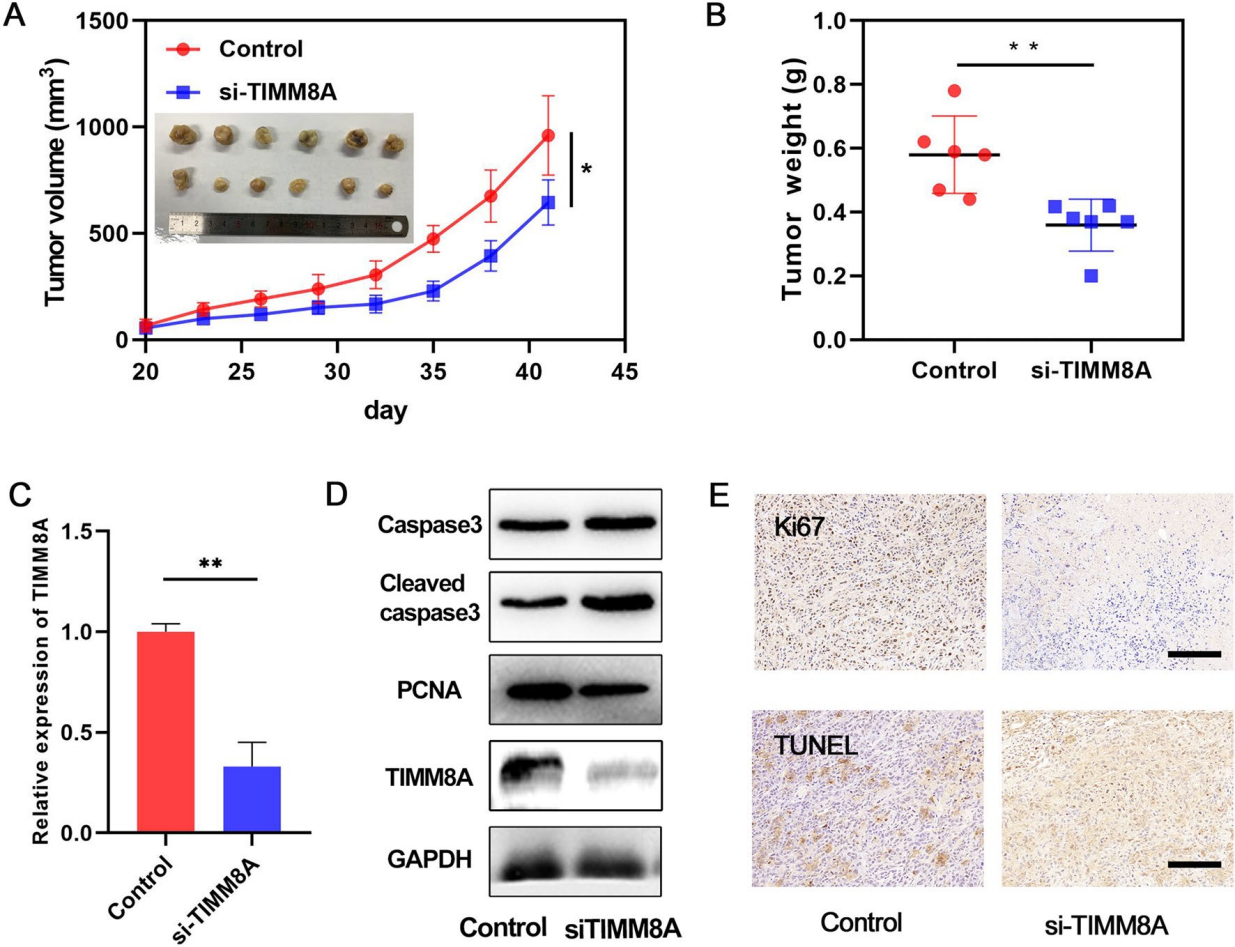
Silencing TIMM8A inhibits tumor growth in vivo. **A** The tumor growth curves and the tumor pictures (insert) (*p < 0.05). **B** The tumor weights of control and TIMM8A inhibited groups (**p < 0.01). **C** Relative expression of TIMM8A mRNA in control and TIMM8A inhibited groups (**p < 0.01). **D** The expression levels of Caspase-3, Cleaved caspase-3, PCNA and TIMM8A in control and TIMM8A inhibited groups. **E** The xenograft tumors with Ki67 immunostaining and TUNEL staining (Scar bar = 200 µm)

We selected different types of breast cancer cell lines (MDA-MB-231 and MCF7) as representatives to explore the function of TIMM8A. By CCK8 assay, we found that inhibition of TIMM8A inhibited proliferation of both MDA-MB-231 and MCF7 cells (Fig. 5A). We further verified the effect of TIMM8A on the proliferation of breast cancer cells using EdU assay. The results showed that in both MDA-MB-231 and MCF7 cell lines, the proliferating cells was reduced after the expression of TIMM8A was inhibited, indicating that TIMM8A has a promoting effect on cell proliferation. (Fig. 5B) Next, we used JC-1 reagent to explore the effect of TIMM8A on mitochondrial membrane potential. We found that after the expression of TIMM8A was inhibited, the JC-1 dimers were reduced, indicating that TIMM promotes proliferation by affecting mitochondrial function (Fig. 5C, D). In order to evaluate the effect of TIMM8A on apoptosis of breast cancer cells, we detected the levels of apoptosis-related proteins in different treatment groups by western blot, and the results showed that after the expression of TIMM8A was inhibited, the pro-apoptotic protein Bax was significantly increased, while the anti-apoptotic protein Bcl-2 was decreased, indicating TIMM8A’s ability to inhibit apoptosis (Fig. 5E). Using the patient-derived tumor fragment platform, we assessed the expression of Ki67 and found that Ki67 expression in human breast cancer fragments decreased after incubation with TIMM8A inhibitors for one week (Fig. 5F). These results indicate that silencing TIMM8A can inhibit the proliferation of breast cancer in vitro. Similarly, silencing TIMM8A inhibits tumor growth in the breast cancer PDX mouse model. The tumor growth rate and tumor weight in TIMM8A inhibited group were significantly lower than those in control group (Fig. 6A, B). We further verified the expression levels of TIMM8A mRNA in tumors (Fig. 6C), and detected apoptosis and proliferation related indicators, such as Caspase 3, proliferating cell nuclear antigen (PCNA), and found that the results were consistent with in vitro cell experiments (Fig. 6D). In addition, IHC results showed that Ki67 levels in tumors decreased and TUNEL levels in tumors increased after TIMM8A inhibitors were administered (Fig. 6E), which further indicates that TIMM8A inhibition can suppress the proliferation and growth of breast cancer.

## Discussion

While the biological functions of TIMM family have gradually advanced, the potential role of TIMM8A has never been investigated in cancer. We hope that our studies could facilitate an available knowledge about improvement of treatment strategy and accuracy of prognosis for patients with breast cancer. Therefore, we firstly performed a pan-cancer analysis to investigate the expression of TIMM8A in different cancer types. Then, the expressions of TIMM8A were compared in breast cancer samples and normal tissues in TCGA, GTEx and Human Protein Atlas database, indicating that TIMM8A was highly expressed in breast cancer tissues. This conclusion was, likewise, supported by the patients’ samples from our clinical specimens. And increased TIMM8A were found to be associated with a worse RFS and OS by the Kaplan–Meier survival analysis. Moreover, TIMM8A expression was also significantly correlated with the clinical characteristics of the patients with breast cancer, including clinical stage, T category, distant metastasis. Based on univariate and multivariate Cox regression analyses, TIMM8A was identified as an independent prognostic factor for breast cancer. Most importantly, we verified the biological function of TIMM8A in different models in vivo and in vitro, which can promote tumor proliferation and act as an oncogene in breast cancer.

The TIMM protein families have been reported to be implicated in tumor progression and development [24, 25]. For instance, previous studies discovered that TIMM44 was proposed to be in association with susceptibility to breast cancer [26]. And TIMM50 could predict poor prognosis and promote tumor progression of non-small cell lung cancer patients by ERK signaling [27]. In our research, we revealed the functions of TIMM8A in breast cancer. After TIMM8A knockdown, breast cancer cells proliferation showed to be restrained, apoptosis increased. To investigate the mechanism of TIMM8A in breast cancer, online bioinformatics prediction databases and dual luciferase reporter gene assay were applied to predict the potential target genes of TIMM8A. In this study, circRNA, including hsa_circ_0107314, hsa_circ_0021867 and hsa_circ_0122013, were found to competitively bind with miRNA, including hsa-miR-34c-5p and hsa-miR-449a, thereby weaken the repression from miRNA to the mRNA of TIMM8A.

Indeed, the functional and integrity stage of mitochondria are vital for cell proliferation, apoptosis, and survival [28, 29]. Mitochondrial function is dramatically dependent on the import of cytosolic proteins, and defects in the translocation system can lead to significant functional impairment [30]. TIMM8A is a key component of the TIMM family, which is involved in these functions in breast cancer biology previously described. Considering that TIMM8A functions as an oncogene and that it may have potential applications as a mitochondria-targeted therapy which is a promising anticancer strategy.

## Conclusion

In the current study, we performed the first-ever comprehensive analysis about the expressions, clinical values and biological functions for TIMM8A in breast cancer. All data demonstrate that TIMM8A might be a potential prognostic indicator and treatment targets for breast cancer patients.

## Supplementary Information

**Additional file 1**: **Table S1** High expressed pathways in the result of Gene set enrichment analysis (GSEA). **Table S2** Low expressed pathways in the result of Gene set enrichment analysis (GSEA). **Table S3** Kyoto Encyclopedia of Genes and Genomes (KEGG) pathway analysis and Gene Ontology (GO) annotations of TIMM8A associated proteins. **Table S4** The potential upstream miRNAs of TIMM8A. **Table S5** The candidate circRNAs for hsa-miR-34c-5p. **Table S6** The candidate circRNAs for hsa-miR-449a.

## Data Availability

Data and materials are available upon request.
